# Extraneous Organisms in Vaccine Lymph

**Published:** 1902-06-21

**Authors:** 


### EXTRANEOUS ORGANISMS IN VACCINE LYMPH.

The *Lancet* has issued another report on the various brands of glycerinated calf vaccine lymphs in common use, and although the result of the investigation is to show that some of the lymphs are of a high degree of purity, it is evident that others are exactly the opposite, and one cannot but feel that the tables given are such as to afford good excuse to anyone who may object to being vaccinated with the ordinary calf lymph of commerce. It seems sufficiently evident that the freer the lymph is from extraneous pyogenic and other organisms the less marked will be the inflammatory concomitants of vaccination, and that much of the irritation which has generally been regarded as essential to a successful vaccination is not due to the vaccinal organism but to extraneous organisms. This being so the number of colonies of these other organisms discovered on making cultures of the various samples on Agar plates is a matter of the first importance, and it is distinctly disquieting to find how enormously the different samples vary when brought to this test, ranging as they do from 0 in one sample to 10,000 in another, while in another the colonies were uncountable. It must be stated that the lymph in which no extraneous organisms were discovered (Merck's) seemed to have been so freely glycerinated that the certainty of its action had become impaired, some specimens being quite inert. The next lymph in purity was that of the Local Government Board. This was proved to be active, and 12 colonies were found in cultures from it. The next on the list had 26 colonies; there were six others with less than 100 colonies; and then, as we have said, the numbers rapidly ran up until in one they were marked as "uncountable." In the column giving the number of colonies in the anærobic tube we find Merck's again containing none, and the Local Government Board lymph only 14, while some others are marked "tens of thousands" and "innumerable." These results are very interesting, and the *Lancet* deserves thanks for the care it has taken in the conduct of this investigation. It would appear that the manufacturers of certain lymphs, perhaps in their fear lest they may impair the efficacy of the lymph, are careful to avoid glycerinating it to such an extent as to get rid of all organisms, of which indeed they leave a large number. By these lymphs active local lesions are obtained, but, especially in stout people, the accompanying œdema is very marked, and the constitutional disturbance is considerable. Then there are lymphs in which the glycerination has been well carried out, and the number of organisms present is but small, but the lymph remains sufficiently potent to induce typical vaccine vesicles without giving rise to too violent a reaction. If, when using such lymphs, care be taken to sterilise the skin and prevent access of extraneous germs to the abraded surface, the vaccine reactions, both general and local, run a typical course. Lastly, there are certain lymphs in which the glycerination and dilution have been carried to such a point that "there is only a slight margin between what may be called first class vaccine lymph and lymph which, although pure, is not absolutely trustworthy. As a type of this class Merck's is perhaps the best." Evidently what is wanted is a lymph with all the activity of that supplied by the Local Government Board and all the purity of that furnished by Merck. What we do not want is lymph containing the number of extraneous organisms that are to be found in some of the samples now on the market.

